# Segmental Mandibulectomy and Mandibular Reconstruction with Fibula-Free Flap Using a 3D Template

**DOI:** 10.3390/jpm14050512

**Published:** 2024-05-11

**Authors:** Melania Tatti, Filippo Carta, Mauro Bontempi, Sara Deriu, Cinzia Mariani, Valeria Marrosu, Emanuele Foddis, Clara Gerosa, Giuseppe Marongiu, Luca Saba, Andrea Figus, Massimiliano Pau, Bruno Leban, Roberto Puxeddu

**Affiliations:** 1 Unit of Otorhinolaryngology, Department of Surgery, Azienda Ospedaliero-Universitaria di Cagliari, University of Cagliari, 09100 Cagliari, Italy; m.tatti@aoucagliari.it (M.T.); m.bontempi@aoucagliari.it (M.B.); deriusara26@gmail.com (S.D.); cinzia.mariani@unica.it (C.M.); v.marrosu@aoucagliari.it (V.M.); emafoddis@icloud.com (E.F.); puxeddu@unica.it (R.P.); 2 Unit of Pathology, Department of Medical Sciences and Public Health, Azienda Ospedaliero-Universitaria di Cagliari, University of Cagliari, 09100 Cagliari, Italy; clara.gerosa@unica.it; 3 Unit of Orthopedics and Traumatology, Department of Surgery, Azienda Ospedaliero-Universitaria di Cagliari, University of Cagliari, 09100 Cagliari, Italy; giuseppe.marongiu@unica.it; 4 Department of Science of the Images, Azienda Ospedaliero-Universitaria di Cagliari, University of Cagliari, 09100 Cagliari, Italy; lucasaba@unica.it; 5 Unit of Plastic Surgery, Department of Surgery, Azienda Ospedaliero-Universitaria di Cagliari, University of Cagliari, 09100 Cagliari, Italy; andreafigus@hotmail.com; 6 Department of Mechanical, Chemical and Materials Engineering, University of Cagliari, 09123 Cagliari, Italy; massimiliano.pau@unica.it (M.P.); bruno.leban@unica.it (B.L.); 7 Unit of Otorhinolaryngology, King’s College Hospital London-Dubai, Dubai P.O. Box 340901, United Arab Emirates

**Keywords:** mandibular reconstruction, free flap, 3D reconstruction, oral cancer

## Abstract

Introduction: The present study evaluates the influence of virtual surgical planning with a preoperative 3D resin model on aesthetic and functional outcomes in patients treated by segmental mandibulectomy and reconstruction with fibula-free flap for oral cancer. Methods: All consecutive patients who underwent segmental mandibulectomy and mandibular reconstruction with a fibula-free flap using a 3D template at our department from January 2021 to January 2023 were included in the study. “Patients control” were patients treated by reconstruction with a fibula-free flap without using a 3D template. Three-dimensional modeling was performed by converting from preoperative computed tomography to a stereolithography format to obtain the resin 3D models. Qualitative analysis of anatomical and aesthetic results consisted of the evaluation of the patients’ aesthetic and functional satisfaction and the symmetry of the mandibular contour observed at clinical examination. Quantitative analysis was based on the assessment of the accuracy and precision of the reconstruction by comparing preoperative and postoperative computed tomograms as objective indicators. Results: Seven patients (five males and two females, mean age of 65.1 years) were included in the study. All patients showed a symmetric mandibular contour based on the clinical examination. After recovery, six patients (85.7%) considered themselves aesthetically satisfied. The quantitative analysis (assessed in six/seven patients) showed that the mean difference between preoperative and postoperative intercondylar distance, intergonial angle distance, anteroposterior dimension, and gonial angle improved in the 3D template-assisted group. Conclusion: The 3D-printed template for mandibular reconstruction with microvascular fibula-free flap can improve aesthetic outcomes in comparison with standard approaches.

## 1. Introduction

Oral squamous cell carcinoma (OSCC) is the most common head and neck cancer, with an incidence of 389,846 new cases and 188,438 deaths in 2022 worldwide [1]. Stages III to IV are considered locoregionally advanced [2] and their prognosis is generally poor [3]. The gold standard treatment in patients with OSCC is represented by primary surgery, associated with adjuvant treatments in patients with risk factors [4]. Improvement in quality of life in OSCC survivors is a major clinical challenge, especially in cases of mandibular involvement [5], since segmental mandibulectomy and the subsequent reconstruction have a crucial role in terms of aesthetic and functional outcomes [6]. Different modalities for mandibular reconstruction have been reported but composite-free flaps are considered the best options and nowadays, a fibula-free flap is often the first choice [7]. Usually, the titanium plate used to stabilize the flap at the receiving site is molded intraoperatively before osteotomies directly on the exposed mandible [8]. Virtual surgical planning has been developed to optimize reconstructive outcomes. Preoperative 3D virtual modeling of skulls created using preoperative computed tomography (CT) scans allows the surgeon to model the titanium plate before surgery, simplifying the intraoperative steps and decreasing operative time [8,9,10,11,12]. This protocol was first described in 1993 by Rose et al. [13] and it became a milestone in the history of mandibular reconstruction.

The present study evaluated the impact of virtual surgical planning for free flap reconstruction with preoperative 3D virtual modeling after segmental mandibulectomy in patients with OSCC on both aesthetic and functional outcomes using subjective and objective methods.

## 2. Materials and Methods

The study was approved by the Ethical Committee of our University Hospital (PG/2021/14276). All consecutive patients who underwent segmental mandibulectomy and computed assisted mandibular reconstruction with a fibula-free flap using a 3D template at our department, from January 2021 to January 2023, were included in the study. “Patients control” were patients treated by reconstruction with a fibula-free flap without using a 3D template (12 patients, from January 2019 to December 2020). All patients had a diagnosis of OSCC with clinical evidence of mandibular bone invasion and were “naïve”, without a clinical history of previous head and neck cancer.

Preoperative evaluation included a complete head and neck (HN) examination, fiberoptic image-enhanced endoscopy [14], HN CT, magnetic resonance imaging (MRI), and CT angiography of the lower limbs to evaluate the donor fibular pedicle.

Comorbidities and performance status (PS) were evaluated with the Charlson/Deyo comorbidity score [15] and with the Eastern Cooperative Oncology Group (ECOG) score, [16,17] respectively.

Patients were staged using the eighth edition of the TNM staging system [2] and all cases were discussed by a multidisciplinary team. A comprehensive preoperative speech and swallowing evaluation was performed for all patients.

Three-dimensional models were obtained from a volumetric set of CT scans of the patients involved in the study. Preoperative 1.0-mm thick slices CT of the mandible and maxilla (facial scan) and fibula (with concomitant CT-angiography) were performed at the radiology department of our University Hospital.

The 3D modeling phase is performed at the 3D printing lab of the Orthopedics and Traumatology Unit of our University Hospital.

DICOM (Digital Imaging and Communications in Medicine) files of the CT scans were imported into the segmentation software, Mimics Innovation Suite (MIS) 25.0 (distributed by Materialise, Leuven, Belgium).

The first phase, called segmentation, is needed to remove not-bony parts and to reduce artifacts (scattering and noises) due to the presence of teeth and/or metal implants. Then, a 3D object file of the entire mandibula is exported in the 3D modeling software, Materialise 3-Matic, version 17.0 (Materialise, Leuven, Belgium), to perform further adjustments of the mandibular replica such as smothering of the surface or thickness analysis. Finally, after surgeon approval, the model is exported in an .STL file format, ready for the 3D printer software. Three-dimensional printing is performed via SLA (Stereolithography) printers (Formlab 2, Formlabs^®^, Somerville, MA, USA). The 3D printing process took an average of 8.1 h for each model (range of 7.2–8.4 h), including all post-processing procedures (curing and polishing).

Pre-conformation of fixation plates with the 3D template was always performed by the same surgeon 48–72 h before the surgical procedure and both the fixation plate (MEDICON ImplantArt Osteosyntesis, Medicon Instrumente, CMF Medicon Surgical, Jacksonville, FL, USA) and 3D template were sent to the sterilization center to be accessible in the surgery room the day of surgery.

The mandibular resection was tailored based on tumor extension and classified according to Brown et al. [18] in class I (lateral), class II (hemi mandibulectomy), class III (anterior mandibulectomy), and class IV (extensive) with the addition of “c” in case of condylectomy. Tumor extension to the tongue required segmental mandibulectomy associated with the glossectomy that was classified according to Ansarin et al. [19]. In our series, only class II and class III mandibulectomies were performed and condyles were always preserved. Surgical defects of the oral cavity were primarily reconstructed with microvascular fibula-free flaps which have the advantages of consistent shape, wide length, and distant location that allows a two-team approach at the same time and low donor-site morbidity [20,21,22]. A fibula-free flap inset starts with the fixation of the plate previously modeled, followed by the fitting of the fibular flap. Anastomoses were performed using an operative microscope (ZEISS S7 Microscope, Carl Zeiss, New York, NY, USA; focal length 250 mm). The arterial anastomosis was performed with synthetic nonabsorbable 8/0 or 9/0 nylon sutures. The venous anastomosis was performed with a coupler device (Microvascular Anastomotic Coupling System, Synovis Life Technologies, Minneapolis, MN, USA). All patients received a single bolus of heparin sodium (1500 IU) at least 5 min before the transfer of the flap.

A temporary tracheostomy was performed in all patients to avoid postoperative respiratory distress. A nasogastric feeding tube was inserted and kept in place until an acceptable swallowing function was restored. An Ear, Nose, and Throat resident monitored the free flap every hour during the first 48 h and every 4 h up to 5 postoperative days according to the internal protocol to detect early signs of ischemia requiring surgical exploration/revision of the anastomosis [23]. Postoperative complications were classified according to the Clavien-Dindo system [24]. Speech and swallowing therapy started 10–12 days after surgery in patients with the regular postoperative course.

Patients with risk factors for recurrence underwent adjuvant therapy according to NCCN guidelines [4].

All patients were postoperatively included in our oncological follow-up [25].

Aesthetic results were evaluated with qualitative and quantitative analysis. The qualitative analysis considered the personal aesthetical satisfaction and the symmetry of the mandibular contour at clinical examination. Personal aesthetic satisfaction was expressed by patients as YES or NO. Objective assessments of symmetry were performed by the ENT and the plastic surgeons, according to the “4-point classification” of Katsuragi [26]: 4 (“excellent”), indicating a symmetrical mandibular and cheek outline; 3 (“good”), indicating slight asymmetries, such as depressed cheeks or lip deformities; 2 (“fair”), indicating visible facial scars or an asymmetrical soft tissue outline; and 1 (“poor”), indicating an asymmetrical mandibular outline, an exposed skin island with poor color match, and any other defects.

**Figure 1:**
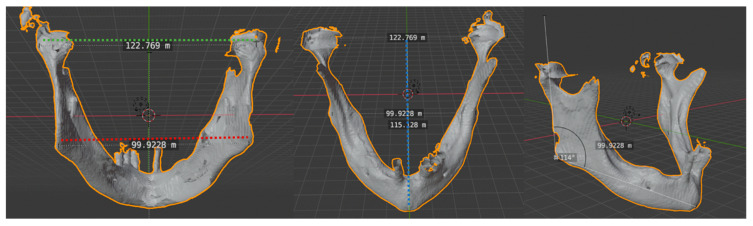
Transverse and anteroposterior dimensions and gonial angle. Intercondylar distance: the distance between the two condylar heads (transverse dimension). Intergonial distance: the distance between the two gonial angles (transverse dimension). Anteroposterior distance: obtained using a perpendicular line drawn from the mandibular midline to the center point of the intercondylar length. Gonial angle: defined as the angle formed by a tangent to the lower border of the mandible and a tangent touching the posterior border of the ramus at two points, one at the condyle and one at the angle region.

Quantitative analysis was based on the assessment of the accuracy and precision of the reconstruction, comparing preoperative and postoperative CT objective indicators, as described by Zhang [27]. Three-dimensional virtual reconstruction of preoperative and postoperative computed tomogram DICOM files was performed with OsiriXLite and Meshmixer. Transverse dimensions, anteroposterior dimension, and the gonial angle were measured and compared (Figure 1).

The accuracy and precision of the reconstruction were evaluated based on the difference between pre- and postoperative parameters.

The evaluation of functional outcomes was based on the recovery of exclusive oral feeding.

The validated and internationally approved European Organization for Research and Treatment of Cancer Quality of Life Questionnaire Version 3 (EORTC QLQ-C30) [28] and the University of Washington Quality of Life (UW-QOL v.4) survey [29] were used to assess postoperative quality of life of our patients.

Statistical analysis was conducted with the T-student test for quantitative variables (unpaired and paired) and with the Chi-Quadro test for qualitative variables.

## 3. Results

Seven patients were included in the present study. All patients’ features and procedures are detailed in Table 1. The fibula-free flap was harvested by the plastic surgeon simultaneously with the resection and the free flap inset was made by both ENT and plastic surgeons after the confirmation of free margins from the frozen section.

Three patients underwent lateral hemi-pelvi-mandibulectomy (class II according to Brown) and four patients underwent anterior pelvi-mandibulectomy (class III according to Brown) [18] associated with type IVa glossectomy according to Ansarin et al. [19] for the extension of tumor to the tongue. All patients underwent synchronous neck dissection, which was bilateral in four patients (57.1%) and unilateral in three patients (42.9%) (see Table 1).

The median operative time was 495.7 min (range of 440–540 min) in the 3D group and 540 min (range of 480–600 min) in the control group.

Margins of resection were free from disease at definitive histology in all our patients.

Qualitative and imaging analysis was obtained in six/seven patients because one patient died one month after surgery from other causes. In the qualitative analysis, six patients (85.7%) considered themselves aesthetically satisfied after reconstructive surgery. All patients (100%) showed a symmetric mandibular contour based on the clinical examination. Objective qualitative and quantitative results are detailed in Table 2 and Table 3, respectively.

Five patients (71.4%) recovered a physiological oral feeding with solid and or semisolid food after rehabilitation. One patient (14.3%), with mandibular defect class III and type IVa glossectomy, slowly achieved oral feeding with soft food but required nutritional support with gastrostomy. One patient (14.3%) with mandibular defect class III and type IVa glossectomy had a worsening general global condition with poor functional outcomes: gastrostomy was needed as the only way of food intake and he died one month after surgery for other causes (Table 4).

Pre- and postoperative EORTC QLQ-C30 questionnaire and the postoperative UW-QOL v.4 questionnaires were available in six out of seven patients (Table 5 and Table 6).

At the last follow-up visit (mean time of 2.01 years, range of 0.3 to 1.67 years), three patients (42.85%) were alive and free of disease, one patient (14.3%) experienced distant metastases and is alive with the disease under systemic treatment, and three patients (42.85%) died for other causes (DOC). The five-year disease-specific survival (DSS), relapse-free survival, and overall survival were 100%, 75%, and 57.1%, respectively, but the DSS observed in our series overestimates the prognosis of patients with advanced OSCC because of the three DOC patients within the first 6 months of follow-up for systemic diseases (one for heart failure, one for hepatic failure, and one for complications of renal disease).

## 4. Discussion

Microvascular surgery in the 1980s revolutionized head and neck reconstruction, with standing microvascular bone-free flaps as the gold standard for the reconstruction of the mandible; nowadays, a fibula-free flap with titanium plate fixation is widely accepted as the first choice because of its thickness, length, and bone uniformity, which make it the ideal support for eventual implants and a good match for the alveolar ridge [7,21,30,31]. The introduction of virtual planning and 3D-printing modeling using preoperative CT data have been introduced to provide more accurate reconstruction and reduce operative time [12,27,32]. The anatomic models can be used preoperatively and intraoperatively, reducing the free flap ischemia time (in the literature, the risk of flap failure is increased with each additional 5 min of ischemia) [33]. In our series, operative time was shorter in patients treated with the aid of the 3D model (495.7 min versus 540 min).

There are three main factors during the preoperative planning that influence the accuracy of the mandibular reconstruction: the extent of the resection of the mandible (which is related to the extent of the tumor and should not be influenced by the reconstructive technique), the modeling of the plate on the 3D model, and the proper setting of the screws [34]. As pointed out by Lu et al. [35], we observed that the pre-bending titanium plate is helpful for restoring a more physiological curve of the mandible. With the aid of the 3D template obtained from high-resolution CT scans, the titanium plate reflects exactly the real preoperative mandibular bone profile, while the plate modeled intraoperatively could be influenced by the presence of different structures such as the periosteum, the muscles, the fascia, and the cancer. Our technique does not change the accuracy of the setting of the screws, which could be further improved by computer-assisted surgery or computer-aided design/computer-aided manufacturing (CAD/CAM) mandibular reconstruction technology that allows osteotomies to be performed before the ischemia of the fibular free flap with the use of cutting guides [33,36,37,38]. Since Hirsch’s description of the technique in 2009 [39], CAD/CAM for mandibular reconstruction has gained popularity due to its reproducibility and its benefits in improving surgeons’ performance and patient satisfaction (both aesthetic and functional). This technique offers the ability to plan osteotomies of both the resection and the donor sites, mirror the unaffected mandible, evaluate the bone plate relationships for the positioning of dental implants, create surgical resection guides, fabricate patient-specific reconstruction plates, and, most importantly, restore correct occlusion [40,41]. However, barriers to the use of this technology include the high costs and the delays in device manufacturing [37,42]. 

The evaluation of the accuracy of the mandibular reconstruction is based on cosmetic and functional outcomes. In the literature, the personal aesthetic satisfaction of patients is considered a key point in the evaluation of aesthetic outcomes [43]. All patients in the study but one were satisfied with the aesthetic outcomes. Katsuragi et al. [26] underlined the importance of objective assessment of aesthetic results and introduced the “four-point classification”. The Katsuragi classification in our series of patients showed a mean score of 3.42 (between good and excellent). Our analysis found a good correlation between the patients’ perceived esthetic outcomes and the specialists’ assessments.

We evaluated the objective accuracy of the mandibular reconstruction using the four indicators described by Zhang et al. [27]. Our data are comparable with those of the computer-assisted surgery group of Zhang et al. [27], who confirmed the positive role of computer-assisted surgery over conventional surgery. We compared the results of our 3D-assisted surgery group with those observed in patients who underwent conventional reconstruction and, notwithstanding the limit represented by the small size of the series, we observed a greater accuracy of reconstruction in the computer-assisted surgery group than in the conventional group. The same results were obtained by Ren et al. [44] and Zheng et al. [45]. In our experience, the most critical parameters that obtained a mean pre/postoperative difference ≥3 were the intercondylar distance and the gonial angle. These results could be explained by the irreversible changes in the physiological mechanical links between masticatory muscles and bone caused by the osteotomies and the mandibular resection.

We observed that the site of the mandibular defect did not influence the accuracy of reconstruction but, despite the good aesthetic results, the resection of the anterior site of the mandible influenced functional outcomes. Different classifications of mandibular defects have been proposed [45,46,47,48] but the most recent is the classification of Brown et al. [18] who distinguished the mandibular defects in a diagrammatic format from class I to class IV, based on their size and complexity. The increased morbidity of the class III mandibular defect in our series was probably due to the associated resection of the tongue. Similar findings were reported by Gonzalez et al. [49] who observed that the extension of resection to soft tissue, the percentage of the resected tongue, and the size of the skin paddle area used for reconstruction were statistically associated with the postoperative need for a gastrostomy tube. Similar results were reported by Atallah et al. [32] in the GETTEC study. These parameters should be considered a short-term indicator of dysphagia to predict the need for postoperative intensive swallowing therapy. In our study, 5 patients (71.43%) achieved exclusive oral feeding after surgery without gastrostomy tube necessity, namely all three patients (100%) with class II mandibular defects and two patients out of four (50%) with class III mandibular defects. One patient with a class III mandibular defect (25%) recovered oral feeding only after a long rehabilitation and temporary intake support with a gastrostomy. One patient (25%) with a class III mandibular defect experienced poor functional outcomes and a long-term gastrostomy was needed (*p* = 0.14, α = 0.05). In our cohort, adjuvant treatment did not influence functional results in the two groups.

Subjective QOL outcomes can be quantified by personal questionnaires. The QOL of head and neck patients is a field of growing interest but the majority of studies are based on general head and neck populations. Zavalishina et al. [43] and Pamias-Romero et al. [50] focused their attention on QOL following mandibular resection and reconstruction. In these studies, QOL assessment was performed using the UW-QOL v.4 questionnaire [29]. In our series, the “functional” postoperative components of EORTC QLQ-C30 had scores > 50 for all subscales and the mean postoperative results were comparable with mean preoperative scores for physical, role, cognitive, and social subscales. The mean postoperative global health/quality of life score was comparable with the preoperative score, confirming that mandibular reconstruction with a 3D template offered a good global quality of life to our patients.

In the present study, according to the literature, we also investigated “oral-specific” fields with the UW-QOL v.4 questionnaire. According to the literature [51,52,53], we observed that patients rated speech, chewing, and swallowing as more important than the other UW-QOL domains. Speech and swallowing domains obtained mean scores of 68.33 ± 22.29 and 68.33 ± 36.56, respectively, while the chewing domain obtained the lowest score of all the UW-QOL subscales (41.67 ± 20.41). This result is probably caused by the delay in prosthetic rehabilitation; in fact, even though prosthetic rehabilitation is not contraindicated after major surgery [54,55,56], this is uncommonly performed due to concerns about placing dental implants for the potential injuries to the fibula flap. In addition, prosthetic rehabilitation is not covered by the national health system of our country.

Although the main limitations of the present study are the small number of patients and the short postoperative follow-up, since long-term changes in condylar head position may be observed [55], it shows the potentiality of the preoperative 3D modeling of the graft.

## 5. Conclusions

The 3D-printed template for the mandibular reconstruction with a fibula-free flap allows for better aesthetic and functional outcomes in comparison with the standard approach, improving the postoperative overall quality of life.

## Figures and Tables

**Table 1:** Patients treated with segmental mandibulectomy and fibula-free flap reconstruction using the 3D template.

Variables		No of Patients (%)
Age (median—range)	65.1 years—48–78 years	
Gender	Male Female Ratio (M/F)	5 (71.4%) 2 (28.6%) 2.5
Primary site of disease	Alveolar ridge/Retromolar trigone Floor of mouth	3 (42.9%) 4 (57.1%)
Tobacco use	Yes No	6 (90%) 1 (10%)
Alcohol excess	Yes No	5 (71.4%) 2 (28.6%)
Tobacco and alcohol use	---	5 (71.4%)
cTNM	cT4aN0M0 cT3N2bM0	6 (85.7%) 1 (14.3%)
Type of mandibular defect according with Brown	Class II Class III	3 (42.9%) 4 (57.1%)
Type of procedure	Anterior segmental pelvi-glosso-mandibulectomy (mandibular defect class III according to Brown + type IVa glossectomy according to Ansarin) Segmental hemi-pelvi-mandibulectomy (mandibular defect class II according to Brown)	4 (57.1%) 3 (42.9%)
Synchronous neck dissection	Unilateral Bilateral	3 (42.9%) 4 (57.1%)
Postoperative complications	Clavien-Dindo IIIA Clavien-Dindo IIIB	1 (14.3%) 1 (14.3%)
pTNM	pT4aN0 pT4aN1 pT4aN2b pT4aN3b (ENE+)	3 (42.9%) 1 (14.3%) 1 (14.3%) 2 (28.5%)

**Table 2:** Katsuragi classification and our results.

	Outline Symmetry, Including Mental Projection (Both Bony and Soft Tissue)	Scarring in the Face (Including Flap Exposure)	Others	Results (Number of Patients and %)
4 (excellent)	Completely symmetric	No scarring	No lip deformity or facial paralysis	3 (42.9%)
3 (good)	Slightly asymmetric	No scarring	Lip deformity/facial paralysis	4 (57.1%)
2 (fair)	Slightly asymmetric	Scarring including exposed skin graft with good color match	Lip deformity/facial paralysis	0
1 (poor)	Asymmetric or Poor mental projection	Exploded skin island, bad color match	Lip deformity/facial paralysis	0

**Table 3:** Mean scores (M)/standard deviations (SD) by different distances in the 3D template-assisted group.

**Domain**	**Pre-Operative** **3D-Group**	**Postoperative** **3D-Group**	**Pre- and Postoperative Absolute Difference ** **3D-Group**
Intercondilar distance (mm)			
M	107.74	108.9	3.02
SD	9.46	10.2	1.56
Intergonial distance (mm)			
M	89.22	90.7	1.84
SD	7.46	6.21	2.11
Antero-posterior distance (mm)			
M	117.8	118.35	2.41
SD	10.9	9.73	1.9
Gonial angle (°)			
M	113	111	3
SD	3.63	5.3	1.8
**Domain**	**Pre-Operative** **No 3D-Group**	**Post-Operative** **No 3D-Group**	**Pre- and Postoperative ** **Absolute Difference ** **No 3D-Group**
Intercondilar distance (mm)			
M	100	100.1	4.1
SD	4.4	4	3.04
Intergonial distance (mm)			
M	84.8	88	5.28
SD	4.4	5.3	4.5
Antero-posterior distance (mm)			
M	122.1	122.1	2.8
SD	7.1	6.3	1.5
Gonial angle (°)			
M	110.5	109.5	5
SD	8.8	6.9	3.7
Statistical analysis (3D vs. No 3D)			
*p* = 0.46	*p* = 0.13	*p* = 0.74	*p* = 0.27
1 − a = 0.95	1 − a = 0.95	1 − a = 0.95	1 − a = 0.95
CI −9.735–7.57	CI −21.79–14.91	CI −4.64–3.96	CI −14.72–10.72

**Table 4:** Details on comparison between anterior and lateral defects.

Site of Primary	Type of Mandibular Defect According with Brown	Type of Procedure	Resection of Tongue	Resection of Skin	Adjuvant Treatment	Functional Results	Aesthetic Results
Floor of the mouth n = 4	Class III	Anterior segmental Pelvi-glosso-mandibulectomy (n = 4)	Type Iva glossectomy according to Ansarin et al. [19] (n = 4)	No (n = 0)	YES n = 2 NO n = 2	No PEG n = 2 Temporary PEG n = 1 Definitive PEG n = 1	Katsuragi classification 4 n = 1 Katsuragi classification 3 n = 3
Alveolar ridge/retromolar trigone n = 3	Class II	Segmental Hemi-pelvi-mandibulectomy (n = 3)	No (n = 0)	No (n = 0)	YES n = 3 NO n = 0	No PEG n = 3 Temporary PEG n = 0 Definitive PEG n = 0	Katsuragi classification 4 n = 2 Katsuragi classification 3 n = 1

**Table 5:** European EORTC QLQ-C30 in a preoperative and postoperative setting. Mean scores (M)/standard deviations (SD) by subscales.

Domain	Pre-Operative	Post-Operative
Function subscales		
Physical function		
M	77.8	78.9
SD	34.4	22.5
Role function		
M	66.7	55.6
SD	51.6	46.7
Emotional function		
M	41.7	80.5
SD	32.9	6.8
Cognitive function		
M	83.3	94.4
SD	25.8	8.6
Social function		
M	80.5	83.3
SD	24.5	18.3
Global health/Quality of Life		
M	52.8	61.1
SD	41	22.1
Symptom subscales/items		
Fatigue	44.4	44.5
M	50.2	32.2
SD		
Nausea/vomiting		
M	0	8.3
SD	0	20.4
Pain		
M	47.2	33.3
SD	42.7	42.2
Dyspnea		
M	38.9	11.1
SD	49.1	17.2
Insomnia		
M	22.2	11.1
SD	34.4	27.1
Appetite loss		
M	33.3	33.3
SD	36.5	42.2
Constipation		
M	38.9	33.3
SD	49.1	36.5
Diarrhea		
M	5.5	0
SD	13.6	0
Financial difficulties		
M	0	27.8
SD	0	44.3

**Table 6:** The University of Washington Quality of Life (UW-QOL v.4) questionnaire in a post-operative setting. Mean scores (M)/standard deviations (SD) by subscales.

Domain	M	SD	Range
Pain	83.33	20.41	50 to 100
Appearance	95.83	10.21	75 to 100
Activity	83.33	25.82	50 to 100
Recreation	79.17	33.23	25 to 100
Swallowing	68.33	22.29	30 to 100
Chewing	41.67	20.41	0 to 50
Speech	68.33	36.56	0 to 100
Shoulder	90	15.49	70 to 100
Taste	61.67	39.71	0 to 100
Saliva	90	15.49	70 to 100
Mood	70.83	24.58	25 to 100
Anxiety	78.33	27.87	30 to 100
Health-related QOL compared to month before having cancer	45.83	18.82	25 to 75
Health-related QOL during the past 7 days	63.33	15.06	40 to 80
Overall QOL during the past 7 days	73.33	10.33	60 to 80

## Data Availability

The data presented in this study are available upon request from the corresponding author.

## References

[1] Ferlay J., Ervik M., Lam F., Laversanne M., Colombet M., Mery L., Piñeros M., Znaor A., Soerjomataram I., Bray F. (2004). Global Cancer Observatory: Cancer Today. Lyon, France: International Agency for Research on Cancer. https://gco.iarc.who.int/today.

[2] Gress D.M., Edge S.B., Green F.L., Washington M.K., Asare E.A., Brierley J.D., Byrd D.R., Compton C.C., Jessup J.M., Winchester D.P., Amin M.B., Edge S.B. (2017). Principles of Cancer Staging. AJCC Cancer Staging Manual.

[3] Tagliabue M., De Berardinis R., Belloni P., Gandini S., Scaglione D., Maffini F., Mirabella R.A., Riccio S., Gioacchino G., Bruschini R. (2022). Oral tongue carcinoma: Prognostic changes according to the updated 2020 version of the AJCC/UICC TNM staging system. Acta Otorhinolaryngol. Ital..

[4] NCCN Clinical Practice Guidelines in Oncology (NCCN Guidelines^®^) Head and Neck Cancers, Version 3.2024—February 29, 2024. https://www.nccn.org/professionals/physician_gls/pdf/head-and-neck.pdf.

[5] Haraldstad K., Wahl A., Andenæs R., Andersen J.R., Andersen M.H., Beisland E., Borge C.R., Engebretsen E., Eisemann M., Halvorsrud L. (2019). A systematic review of quality of life research in medicine and health sciences. Qual. Life Res..

[6] Woliansky J., Green L., Sim F. (2023). Does Segmental Mandibulectomy Involving Critical Functional Sites Affect Quality of Life?. J. Oral Maxillofac. Surg..

[7] Hurley C.M., McConn Walsh R., Shine N.P., O’Neill J.P., Martin F., O’Sullivan J.B. (2023). Current trends in craniofacial reconstruction. Surgeon.

[8] Ma H., Shujaat S., Bila M., Sun Y., Vranckx J., Politis C., Jacobs R. (2021). Computer-assisted versus traditional freehand technique for mandibular reconstruction with free vascularized fibular flap: A matched-pair study. J. Plast. Reconstr. Aesthetic Surg..

[9] Jones E.A., Huang A.T. (2023). Virtual Surgical Planning in Head and Neck Reconstruction. Otolaryngol. Clin. N. Am..

[10] Baecher H., Hoch C.C., Knoedler S., Maheta B.J., Kauke-Navarro M., Safi A.F., Alfertshofer M., Knoedler L. (2023). From bench to bedside—Current clinical and translational challenges in fibula free flap reconstruction. Front. Med..

[11] Crosetti E., Succo G., Battiston B., D’Addabbo F., Tascone M., Maldi E., Bertotto I., Berrone M. (2022). Surgical Margins After Com-puter-Assisted Mandibular Reconstruction: A Retrospective Study. Front. Oral Health.

[12] Annino D.J., Hansen E.E., Sethi R.K., Horne S., Rettig E.M., Uppaluri R., Goguen L.A. (2022). Accuracy and outcomes of virtual surgical planning and 3D-printed guides for osseous free flap reconstruction of mandibular osteoradionecrosis. Oral Oncol..

[13] Rose E.H., Norris M.S., Rosen J.M. (1993). Application of high-tech three-dimensional imaging and computer-generated models in complex facial reconstructions with vascularized bone grafts. Plast. Reconstr. Surg..

[14] Ansari U.H., Wong E., Smith M., Singh N., Palme C.E., Smith M.C., Riffat F. (2019). Validity of narrow band imaging in the detection of oral and oropharyngeal malignant lesions: A systematic review and meta-analysis. Head Neck.

[15] Deyo R.A., Cherkin D.C., Ciol M.A. (1992). Adapting a clinical comorbidity index for use with ICD-9-CM administrative databases. J. Clin. Epidemiol..

[16] Oken M.M., Creech R.H., Tormey D.C., Horton J., Davis T.E., McFadden E.T., Carbone P.P. (1982). Toxicity and response criteria of the Eastern Cooperative Oncology Group. Am. J. Clin. Oncol..

[17] Azam F., Latif M.F., Farooq A., Tirmazy S.H., AlShahrani S., Bashir S., Bukhari N. (2019). Performance Status Assessment by Using ECOG (Eastern Cooperative Oncology Group) Score for Cancer Patients by Oncology Healthcare Professionals. Case Rep. Oncol..

[18] Brown J.S., Barry C., Ho M., Shaw R. (2016). A new classification for mandibular defects after oncological resection. Lancet Oncol..

[19] Ansarin M., Bruschini R., Navach V., Giugliano G., Calabrese L., Chiesa F., Medina J.E., Kowalski L.P., Shah J.P. (2019). Classification of GLOSSECTOMIES: Proposal for tongue cancer resections. Head Neck.

[20] Chatterjee D., Rahman Z., Hardsha K.N., Sharma J., Rai R., Menon A. (2022). Reconstruction of complex oromandibular defects by four differ-ent modifications of free fibula osteomyocutaneous flap: A prudent alternative to multiple flaps. J. Plast. Reconstr. Aesthet. Surg..

[21] Awad M.E., Altman A., Elrefai R., Shipman P., Looney S., Elsalanty M. (2019). The use of vascularized fibula flap in mandibular re-construction: A comprehensive systematic review and meta-analysis of the observational studies. J. Craniomaxillofac. Surg..

[22] Wei F.C., Seah C.S., Tsai Y.C., Liu S.J., Tsai M.S. (1994). Fibula osteoseptocutaneous flap for reconstruction of composite mandibular defects. Plast. Reconstr. Surg..

[23] Carta F., Quartu D., Mariani C., Tatti M., Marrosu V., Gioia E., Gerosa C., Zanda J.S.A., Chuchueva N., Figus A. (2020). Compartmental Surgery with Microvascular Free Flap Reconstruction in Patients with T1-T4 Squamous Cell Carcinoma of the Tongue: Analysis of Risk Factors, and Prognostic Value of the 8th Edition AJCC TNM Staging System. Front. Oncol..

[24] Ebner J.J., Mehra T., Gander T., Schumann P., Essig H., Zweifel D., Rücker M., Slankamenac K., Lanzer M. (2019). Novel application of the Clavien-Dindo classification system and the comprehensive complications index® in microvascular free tissue transfer to the head and neck. Oral Oncol..

[25] De Felice F., Lei M., Oakley R., Lyons A., Fry A., Jeannon J.P., Simo R., Guerrero Urbano T. (2021). Risk stratified follow up for head and neck cancer patients—An evidence based proposal. Oral Oncol..

[26] Katsuragi Y., Kayano S., Akazawa S., Nagamatsu S., Koizumi T., Matsui T., Onitsuka T., Yurikusa T., Huang W.C., Nakagawa M. (2011). Mandible reconstruction using the calcium-sulphate three-dimensional model and rubber stick: A new method, ‘mould technique’, for more accurate, efficient and simplified fabrication. J. Plast. Reconstr. Aesthet. Surg..

[27] Zhang L., Liu Z., Li B., Yu H., Shen S.G., Wang X. (2016). Evaluation of computer-assisted mandibular reconstruction with vascularized fibular flap compared to conventional surgery. Oral Surg. Oral Med. Oral Pathol. Oral Radiol..

[28] Bjordal K., De Graeff A., Fayers P.M., Hammerlid E., Van Pottelsberghe C., Curran D., Ahlner-Elmqvist M., Maher E.J., Meyza J.W., Brédart A. (2000). A 12 country field study of the EORTC QLQ-C30 (version 3.0) and the head and neck cancer specific module (EORTC QLQ-H&N35) in head and neck patients. EORTC Quality of Life Group. Eur. J. Cancer.

[29] Hassan S.J., Weymuller E.A. (1993). Assessment of quality of life in head and neck cancer patients. Head Neck.

[30] Molteni G., Gazzini L., Sacchetto A., Nocini R., Comini L.V., Arietti V., Locatello L.G., Mannelli G. (2023). Mandibular reconstruc-tion in head and neck cancer: Which is the gold standard?. Eur. Arch. Otorhinolaryngol..

[31] Dunlap Q., Hairston H., Gardner J.R., Hagood J., Turner M., King D., Sunde J., Vural E., Moreno M.A. (2023). Comparing donor site morbidity in osteocutaneous radial forearm versus fibula free flap for mandibular reconstruction. Am. J. Otolaryngol..

[32] Atallah S., Bozec A., Ransy P., Davrou J., Longis J., Humbert M., Brenet E., Schultz P., Damecourt A., Lacau Saint Guily J. (2021). Functional evaluation of mandibular reconstruction with bone free flap. A GETTEC study. Eur. Ann. Otorhinolaryngol. Head Neck Dis..

[33] Nyirjesy S.C., Heller M., Von Windheim N., Gingras A., Kang S.Y., Ozer E., Agrawal A., Old M.O., Seim N.B., Carrau R.L. (2022). The role of computer aided design/computer assisted manufacturing (CAD/CAM) and 3- dimensional printing in head and neck oncologic surgery: A review and future directions. Oral Oncol..

[34] Chen J., Zhang R., Liang Y., Ma Y., Song S., Jiang C. (2021). Deviation Analyses of Computer-Assisted, Template-Guided Mandibular Reconstruction with Combined Osteotomy and Reconstruction Pre-Shaped Plate Position Technology: A Comparative Study. Front. Oncol..

[35] Lu T., Shao Z., Liu B., Wu T. (2020). Recent advance in patient-specific 3D printing templates in mandibular reconstruction. J. Mech. Behav. Biomed Mater..

[36] Mazzola F., Smithers F., Cheng K., Mukherjee P., Hubert Low T.H., Ch’ng S., Palme C.E., Clark J.R. (2020). Time and cost-analysis of virtual surgical planning for head and neck reconstruction: A matched pair analysis. Oral Oncol..

[37] Weitz J., Grabenhorst A., Singer H., Niu M., Grill F.D., Kamreh D., Claßen C.A.S., Wolff K.D., Ritschl L.M. (2023). Mandibular recon-structions with free fibula flap using standardized partially adjustable cutting guides or CAD/CAM technique: A three- and two-dimensional comparison. Front. Oncol..

[38] Moe J., Foss J., Herster R., Helman J., Ward B.B., VanKoevering K. (2021). An In-House Computer-Aided Design and Computer-Aided Manufacturing Workflow for Maxillofacial Free Flap Reconstruction is Associated with a Low Cost and High Accuracy. J. Oral Maxillofac. Surg..

[39] Hirsch D.L., Garfein E.S., Christensen A.M., Weimer K.A., Saddeh P.B., Levine J.P. (2009). Use of Computer-Aided Design and Computer-Aided Manufacturing to Produce Orthognathically Ideal Surgical Outcomes: A Paradigm Shift in Head and Neck Reconstruction. J. Oral Maxillofac. Surg..

[40] van Baar G.J.C., Forouzanfar T., Liberton N.P.T.J., Winters H.A.H., Leusink F.K.J. (2018). Accuracy of computer-assisted surgery in mandibular reconstruction: A systematic review. Oral Oncol.

[41] Tarsitano A., Battaglia S., Ricotta F., Bortolani B., Cercenelli L., Marcelli E., Cipriani R., Marchetti C. (2018). Accuracy of CAD/CAM mandibular reconstruction: A three-dimensional, fully virtual outcome evaluation method. J Craniomaxillofac Surg.

[42] Vranckx J.J., Desmet O., Bila M.M., Wittesaele W., Wilssens N., Poorten V.V. (2023). Maxillomandibular Reconstruction Using Insourced Virtual Surgical Planning and Homemade CAD/CAM: A Single-Center Evolution in 75 Patients. Plast. Reconstr. Surg..

[43] Zavalishina L., Karra N., Zaid W.S., El-Hakim M. (2014). Quality of life assessment in patients after mandibular resection and free fibula flap reconstruction. J. Oral Maxillofac. Surg..

[44] Ren W., Gao L., Li S., Chen C., Li F., Wang Q., Zhi Y., Song J., Dou Z., Xue L. (2018). Virtual Planning and 3D printing modeling for mandibular reconstruction with fibula free flap. Med. Oral Patol. Oral Cir. Bucal..

[45] Zheng L., Lv X., Zhang J., Liu S., Zhang J., Zhang Y. (2018). Translating Computer-Aided Design and Surgical Planning Into Successful Mandibular Reconstruction Using a Vascularized Iliac-Crest Flap. J. Oral Maxillofac. Surg..

[46] Pavlov B.L. (1974). Klassifikatsiia defektov nizhnei cheliusti [Classification of mandibular defects]. Stomatologiia.

[47] Jewer D.D., Boyd J.B., Manktelow R.T., Zuker R.M., Rosen I.B., Gullane P.J., Rotstein L.E., Freeman J.E. (1989). Orofacial and mandibular reconstruction with the iliac crest free flap: A review of 60 cases and a new method of classification. Plast. Reconstr. Surg..

[48] Urken M.L., Weinberg H., Vickery C., Buchbinder D., Lawson W., Biller H.F. (1991). Oromandibular reconstruction using microvascular composite free flaps. Report of 71 cases and a new classification scheme for bony, soft-tissue, and neurologic defects. Arch. Otolaryngol. Head Neck Surg..

[49] Gonzalez S.R., Hobbs B., Vural E., Moreno M.A. (2019). Functional outcome predictors following mandibular reconstruction with osteocutaneous fibula free flaps: Correlating early postoperative videofluoroscopic swallow studies with long-term clinical results. Maxillofac. Plast. Reconstr. Surg..

[50] Pamias-Romero J., Saez-Barba M., de-Pablo-García-Cuenca A., Vaquero-Martínez P., Masnou-Pratdesaba J., Bescós-Atín C. (2023). Quality of Life after Mandibular Reconstruction Using Free Fibula Flap and Customized Plates: A Case Series and Comparison with the Literature. Cancers.

[51] Hsing C.Y., Wong Y.K., Wang C.P., Wang C.C., Jiang R.S., Chen F.J., Liu S.A. (2011). Comparison between free flap and pectoralis ma-jor pedicled flap for reconstruction in oral cavity cancer patients-a quality of life analysis. Oral Oncol..

[52] Rogers S.N., Laher S.H., Overend L., Lowe D. (2002). Importance-rating using the University of Washington quality of life questionnaire in patients treated by primary surgery for oral and oro-pharyngeal cancer. J. Craniomaxillofac. Surg..

[53] Zavala A., Al Deek N.F., Chang Y.M., Tsai C.Y., Wei F.C. (2021). Reconstruction of mandibular defects involving the central segment with fibula osteoseptocutaneous free flap following ameloblastoma resection: Patient-reported outcomes. J. Plast. Reconstr. Aesthet. Surg..

[54] Sandoval M.L., Rosen E.B., Robert A.J., Nelson J.A., Matros E., Gelblum D.Y. (2020). Immediate dental implants in fibula free flaps to reconstruct the mandible: A pilot study of the short-term effects on radiotherapy for patients with head and neck cancer. Clin. Implant Dent. Relat. Res..

[55] Khatib B., Cheng A., Sim F., Bray B., Patel A. (2020). Challenges with the Jaw in a Day Technique. J. Oral Maxillofac. Surg..

[56] Ong A., Williams F., Tokarz E., Shokri T., Hammer D., Ducic Y. (2021). Jaw in a Day: Immediate Dental Rehabilitation during Fibula Reconstruction of the Mandible. Facial Plast. Surg..

